# Case analysis of hepatotoxicity caused by vancomycin

**DOI:** 10.1186/s13256-024-04574-4

**Published:** 2024-06-04

**Authors:** Jiayao Wu, Yulu Zhou

**Affiliations:** 1 Department of Pharmacy, Hunan Provincial Maternal and Child Health Hospital, Changsha, China; 2 Department of Pharmacy, Tianjin Medical University General Hospital, Tianjin, China

**Keywords:** Vancomycin, Liver toxicity, Liver injury, Abnormal liver function, Blood concentration, Case analysis

## Abstract

**Background:**

Although the correlation between liver toxicity and vancomycin is generally considered low, it has been observed that the use of vancomycin can lead to abnormal liver function indicators, such as elevated aspartate aminotransferase, alanine aminotransferase, alpha fetoprotein, and jaundice. To further understand the clinical features associated with vancomycin-induced liver toxicity and to provide clinical guidance, we conducted an analysis of the characteristics and clinical manifestations of vancomycin-induced liver injury.

**Methods:**

Patients with liver function injury who received vancomycin treatment at the Third Xiangya Hospital of Central South University and Hunan Maternal and Child Health Hospital between 2016 and 2021 were selected for retrospective analysis of their general characteristics, vancomycin course, dose, liver function index, severity of liver injury, and concomitant medications.

**Results:**

Of the 4562 patients who received vancomycin, 17 patients were finally included, with an incidence rate of 0.37%. Of these patients, 12 were male (70.6%) and 5 were female (29.4%), ranging in age from 17 to 84 years with a mean average age of 45.41 ± 20.405 years. All patients were evaluated using Naranjo’s score, with score ≥ 3. The dosage, time, and plasma concentration of vancomycin were analyzed and it was found that nine patients (52.94%) had abnormal liver function when initially given a dose of 1 g every 12 hours. In total, 14 patients (82.35%) with liver injury were taking vancomycin in combination with two to four drugs, and severe liver injury occurred in patients taking vancomycin in combination with two drugs. The occurrence time of liver injury was 2–12 days after starting vancomycin, with a mean of 4.53 ± 2.401 days. Of these patients, 16 patients (94.1%) showed liver function abnormalities within 7 days of taking the drug, and 2 patients with grade 3–4 liver injury both showed liver function abnormalities within 3 days of taking the drug. Only 4 of the 17 patients (23.53%) had vancomycin blood concentrations within the normal range, and there was no correlation found between blood concentration and severity of liver injury. Analysis of the correlation between the severity of liver injury and vancomycin showed that none of the patients had allergies such as rash, two patients (11.76%) had jaundice, and fatigue occurred in five patients (29.41%). The remaining ten patients (58.82%) had no symptoms related to liver injury. All 17 patients had abnormal aspartate aminotransferase/alanine aminotransferase levels and 9 patients also had abnormal bilirubin levels. In 15 patients (88.24%), the severity of liver injury was grade 1, indicating mild liver injury, and no correlation was observed between the severity of liver injury and creatinine. Of the 17 patients, 1 patient received no intervention, 4 patients stopped taking vancomycin after developing liver injury, 1 patient reduced the dose, and 11 patients (64.7%) were treated with hepatic protectant.

**Conclusion:**

Although the study concluded that the incidence of liver injury was not high, the liver toxicity of vancomycin should still be considered and liver function indicators should be monitored during the clinical use of vancomycin.

A preprint has previously been published [1].

## Background

Various drugs can cause injury, including antibiotics, antidiabetic drugs, antipsychotic drugs, antitubercular drugs, and antineoplastic drugs. Among these, antibiotics have been shown to be the most common agent involved in adverse drug reactions in hospitalized patients, with effects ranging from gastrointestinal to organ dysfunction and hypersensitivity reactions [2]. Vancomycin is a glycopeptide antibiotic that is primarily active against Gram-positive bacteria, such as *Staphylococcus aureus*, *Streptococcus*, *Clostridium*, *Actinomycetes*, *Enterococcus*, *Listeria*, and so on. It does not show cross-resistance with other antibiotics. It exerts antibacterial effects by inhibiting the synthesis of bacterial cell wall glycopeptides, altering the permeability of the bacterial cell membrane and destroying its barrier function, and selectively inhibiting the biosynthesis of bacterial RNA to prevent bacterial replication [3]. Studies have reported that the incidence of adverse events in the vancomycin group was 12.5%, and the most common adverse reactions were nephrotoxicity, ototoxicity, red man syndrome, allergic reactions, and abnormal liver function indicators, such as abnormal aspartate aminotransferase (AST), alanine aminotransferase (ALT), and alpha-fetoprotein (AFP) level [4]. Owing to the excretion of more than 80% of vancomycin in the urine in the form of prototype [5], its nephrotoxicity has also received widespread attention. Although the correlation between liver toxicity and vancomycin is very low, abnormal liver function indicators, such as elevated AST, ALT, AFP, and jaundice, have been observed with the use of vancomycin. A meta-analysis of 20 randomized controlled trial (RCT) studies from 1950 to 2010 showed that 321 patients (6.83%) treated with vancomycin experienced liver function abnormalities, which were mainly mild to moderate increases in serum transaminases [6]. To further investigate the clinical characteristics of vancomycin liver toxicity, we analyzed the clinical data of patients with vancomycin-induced liver injury.

## Methods

### Data source

A total of 4562 hospitalized patients who received vancomycin treatment in the Third Xiangya Hospital from 2016 to 2021 were selected. According to the inclusion and exclusion criteria, 17 patients were finally included. Inclusion criteria: (1) received vancomycin treatment during hospitalization, (2) blood concentration of vancomycin was monitored during medication, (3) had a Naranjo score of ≥ 3 with vancomycin. Exclusion criteria: patients who did not meet the above inclusion criteria.

### Methods

Retrospective research methods were used to read and sort the patient records, and to extract information, such as sex, age, site of infection, vancomycin dosage, blood concentration, adverse reaction time, symptoms, indicators, treatment measures, and outcomes.

### Observation indicators

The purpose of using vancomycin in enrolled patients; time of adverse reactions; symptoms; liver function indicators: AST, ALT, direct bilirubin, indirect bilirubin; renal function: creatinine; plasma concentration; outcomes; and concurrent diseases and medications.

### Statistical methods

SPSS 22.0 was used for statistical analysis.

## Results

### General situation

A total of 17 patients with liver injury were included with an incidence rate of 0.37%, including 12 males (70.6%) and 5 females (29.4%) aged 17–84 years with an average age of 45.41 ± 20.405 years. Among the 17 patients, 1 patient had a history of cholecystectomy, 1 patient had a history of hepatitis B, and 1 patient had a history of alcohol consumption. Overall, 6 patients had aggravated liver injury and the remaining 11 patients had new-onset liver dysfunction. Naranjo’s assessment scale was used to evaluate all patients for the correlation of adverse reactions and score ≥ 3, which was very likely relevant in ten patients and possibly relevant in seven patients. Physicians discontinued vancomycin in all patients. The general information of the patients is presented in Table 1, and the clinical information of the patients is presented in Table 2.

**Table 1:** Patient characteristics

Characteristics	Stratification	Cases (*n* = 17)	No. (%)
Age (years)			
	< 18	1	5.88
	≥ 18 to < 41	8	47.06
	≥ 41 to < 66	5	29.41
	≥ 66	3	17.65
Site of infection			
	Lung	11	64.71
	Intracranial	1	
	Urinary tract	1	
	Postoperative prevention	1	
	Pulmonary infection with bacteremia	1	
	Intracranial infection with bacteremia	1	
Concomitant disease (species)			
	1–3	12	70.59
	4–6	4	23.53
	7–9	1	5.88
Purpose of medication			
	Surgical prophylaxis	1	5.88
	Empiric medication	14	82.35
	Targeted treatment	2	11.77
Naranjo’s score			
	3	1	5.88
	4	6	35.30
	5	6	35.30
	6	4	23.53

**Table 2:** Clinical characteristics of cases of vancomycin hepatotoxicity

Patient no.	Sex	Age (years)	Past history^a^	Purpose of medication: infected site	Comorbidity	Dose	Dosing frequency	Blood concentration^b^ (μg/ml)	Time from medication to liver injury (days)	Total course of treatment (days)	Combination therapy	Symptom	Clinical manifestations of liver damage	Grading of severity of liver damage	Intervention^c^	Outcome	Naranjo’s score (fraction)	
1	Male	17	No	Intracranial, bacteremia	Multiple head trauma	1 g 1 g	Every 12 hours Every 8 hours	1.94	6 (Worsening)	4	Meropenem Linezolid	Swelling of the head and face, bleeding in the nose and mouth, abdominal pain, and repeated fever	AST/ALT increased	1	Glutathione 0.6 g per day	Remission	5	Probable
2	Male	40	No	Unclear, preventive medication after surgery	Femoral neck fracture	1 g	Every 12 hours	4.93	12	12	Perindopril Nifedipine Carvedilole	No obvious symptoms	AST/ALT increased	1	No intervention	Remission	3	Possible
3	Female	51	No	Lung	Chronic myeloid leukemia	1 g	Every 12 hours	37.19	3 (worsening)	3	Metoprolol	Fatigue, fever, drowsiness, and flaky ecchymoses under the skin in many places all over the body	AST/ALT increased	1	Glutathione 1.2 g once per day	Died	4	Possible
4	Female	37	No	Lung	Acute respiratory distress syndrome	0.5 g	Every 12 hours	10.3	4 (worsening)	8	Oseltamivir Methyl prednisolone Metoprolol	Cough and sputum with difficulty breathing, coma	AST/ALT increased	1	Glutathione 1.2 g once per day	Died	4	Possible
5	Male	38	No	Lung	Lymphoma	1 g	Every 12 hours	11.7	3 (worsening)	7	Teicoplanin Trimethazidine	Repeated fever accompanied by dry cough, flushing complexion, and red throat	Bilirubin increased	1	Glutathione 2.4 g once per day	Not relieved	4	Possible
6	Male	19	No	Urinary tract	Acute leukemia	1 g	Every 12 hours	1.2	5	8	Acyclovir Moxifloxacin Imipenem/cilastatin sodium Arsenic trioxide Retinoic acid Hydroxyurea	Fever, fatigue	AST/ALT increased	1	Glutathione 2.4 g once per day Polyene phosphatidyl choline 465 mg once per day	Remission	6	Probable
7	Male	22	No	Lung	Acute myeloid leukemia	1 g	Every 12 hours	2.57	3 (Worsening)	3	Meropenem Caspofungin Valacyclovir Cytarabine Methotrexate Decitabine	Fever, fatigue, and poor appetite	AST/ALT increased	1	Glutathione 2.4 g once per day	Remission	5	Probable
8	Male	62	No	Lung, bacteremia	Leiomyosarcoma	1 g	Every 12 hours	10.58	7	15	Meropenem Fluconazole Piperacillin–tazobactam Telmisartan	Fever	AST/ALT increased	1	Glutathione 2.4 g once per day	Remission	4	Possible
9	Male	19	No	Lung	Acute lymphocytic leukemia	1 g	Every 12 hours	25.7	3 (Worsening)	3	Meropenem Voriconazole Valacyclovir Teicoplanin Ruxitinib Tacrolimus Cyclophosfamide	Fever	AST/ALT/ bilirubin increased	1	Magnesium isoglycyrrhizinate 150 mg once per day Glutathione 2.4 g once per day	Remission	5	Probable
10	Male	39	Drinking history	Intracranial, multiple site infection	Cystic space	0.5 g 1.0 g	Every 8 hours	7.5	4	5 3	Ganciclovir Meropenem Rifampicin Isoniazid	Repeated fever	AST/ALT/bilirubin increased	1	Glutathione 2.4 g once per day	Remission	4	Possible
11	Female	71	Hepatitis B for 1 year	Lung	Cerebral hemorrhage (left ventricle)	1 g	Every 12 hours Every 8 hours	28.9	3	6	Meropenem Entecavir Hydroxychloroquine Oxiracetam Teicoplanin	Repeated fever	AST/ALT increased	1	Withdrawal	Remission	6	Probable
12	Male	48	No	Lung	Acute heart failure	1 g	Every 12 hours Every 8 hours	10.47	3	7	Caspofungin Imipenem/cilastatin sodium	Whole-body skin jaundice	AST/ALT/bilirubin increased	3	Glutathione 2.4 g once per day Polyene phosphatidyl choline 465 mg once per day Ademetionine 1000 mg once per day	Remission	4	Possible
13	Female	34	No	Lung	Acute lymphocytic leukemia	1 g	Every 12 hours Every 8 hours	7.4	3	5	Meropenem Peramivir Valacyclovir Amphotericin B	Fever, poor appetite, and fatigue	AST/ALT increased	1	Withdrawal	Remission	5	Probable
14	Female	56	Cholecystectomy 10 years ago	Lung	Stomach cancer	1 g	Every 12 hours	26.4	4	7	Meropenem	Fatigue, poor appetite	AST/ALT/bilirubin increased	1	Withdrawal	Remission	5	Probable
15	Male	84	Multiple hepatic cysts	Lung	Cerebral infarction, type II respiratory failure, acute exacerbation of COPD	1 g 0.5 g 0.5 g	Every 12 hours Every 8 hours Every 12 hours	20.33	4 (Worsening)	10	Ambroxol Doxofylline	Fever	AST/ALT increased	1	Dose reduction	Remission	6	Probable
16	Male	63	No	Lung	Non-Hodgkin’s lymphoma	1 g	Every 12 hours	17.3	3	5	Meropenem Metoprolol	Fever, jaundice	Bilirubin increased	4	Polyene phosphatidyl choline 465 mg once per day Ademetionine 1000 mg once per day	Died	5	Probable
17	Male	72	No	Wound infection after mitral and tricuspid valve surgery, lung	Valvular heart disease	1 g 0.5 g	Every 8 hours Every 12 hours	30.136	5	8	Cefoperazone sodium/sulbactam sodium Warfarin Quetiapine Nifedipine	Fever	AST/ALT increased	1	Withdrawal	Remission	6	Probable

^a^Is there any liver disease in the past, such as hepatitis

^b^Blood concentration of vancomycin, standard value: 10–15 μg/ml

^c^Whether to stop and protect liver treatment drugs

^d^Naranjo’s score: an adverse reaction assessment scale that assesses whether there is a causal relationship between a drug and an adverse event. An association was defined on the basis of a score of 10 choices, score ≥ 9: a certain causal relationship between the drug and the adverse event; 5 ≤ score < 8: a probable correlation was considered; 1 ≤ score < 4: a possible correlation was considered; and score 0: association was incidental or almost nonexistent.

### Analysis of dosage, time, and plasma concentration of vancomycin in patients with liver injury

#### Vancomycin dosage and treatment course

Among the 17 patients, 9 patients (52.94%) were given vancomycin at a dose of 1 g every 12 hours, among which four patients had higher-than-normal blood concentrations of vancomycin, and one patient had liver injury severity of grade 4; the severity of liver injury was grade 1 in three cases, which was lower than normal. In total, four patients (23.53%) were given 1 g every 8 hours after the initial administration of 1 g every 12 hours. Out of these four patients, the blood concentration of vancomycin was higher than the normal value in one patient, two patients were lower than the normal range, and one case had a normal range with grade 3 liver injury severity. The dose adjustment of all patients was based on their vancomycin blood concentration. The average administration time was 6.82 ± 3.264 days, ranging from 3 to 15 days. There were four patients (23.53%) who received vancomycin for 3–6 days, and one of them had grade 4 liver injury. Overall, six patients (35.28%) received vancomycin for 6–9 days, and one case had grade 3 liver injury (Table 3).

**Table 3:** Analysis of vancomycin use in patients with liver injury

Results	Stratification	Cases (*n* = 17)	No. (%)
Dosage			
	1 g every 12 hours	9	52.94
	1 g every 12 hours → 1 g every 8 hours	4	23.53
	1 g every 8 hours → 0.5 g every 12 hours	1	
	1 g every 12 hours → 0.5 g every 8 hours → 0.5 g every 12 hours	1	
	0.5 g every 12 hours	1	
	0.5 g every 8 hours → 1.0 g every 8 hours	1	
Dosing days (days)			
	≤ 3	3	17.65
	3 < days ≤ 6	4	23.53
	6 < days ≤ 9	6	35.28
	9 < days ≤ 12	2	11.77
	12 < days ≤ 15	2	11.77
Concomitant medication (types)			
	1	2	11.77
	2	5	29.41
	3	5	29.41
	4	4	23.53
	5	1	5.88
Time to liver damage after medication (days)			
	3 days inside	8	47.1
	3–7 days	8	47.1
	> 7 days	1	

#### Concomitant medication

The study examined the effect of drug combination in 17 patients. The mean number of concomitant medications was 2.82, and 14 patients (82.35%) used two to four drugs at the same time (Table 3), of which five patients (29.41%) used two drugs, another five patients (29.41%) used three drugs, and four patients (23.53%) used four drugs; two patients with grade 3–4 liver injury were treated with two drugs at the same time.

#### The time of liver damage after medication

The time of liver damage occurrence in 17 patients was analyzed. Abnormal liver function indexes appeared within 2–12 days after patients began taking vancomycin, with an average onset of 4.41 ± 2.293 days. In total, 16 patients (94.1%) showed abnormal liver function within 7 days after drug administration (Table 3), 1 patient had abnormal liver function 12 days after treatment, and 2 patients had grade 3–4 liver injury 3 days after treatment.

#### Vancomycin plasma concentration

After vancomycin administration, the plasma concentration of all patients was determined by high-performance liquid chromatography (HPLC). The blood drug concentration ranged from 1.20 to 37.19 μg/ml, with an average of 14.8906 ± 11.21257 μg/ml. Among them, six patients (35.29%) did not reach effective blood concentration. Overall, seven patients (41.18%) had a vancomycin level beyond the normal range. Among these seven patients, six patients (85.71%) had a Naranjo’s score indicating a possible reaction, and one case had a probable reaction. However, only one patient with grade 3–4 liver function damage had a blood drug concentration above the upper limit of normal, which is grade 4 liver function damage, suggesting that there may be no correlation between severity and plasma concentration.

### Correlation analysis between the severity and vancomycin

#### Whether there is drug rash with eosinophilia and systemic symptoms (DRESS) syndrome

DRESS syndrome, also known as drug hypersensitivity syndrome or drug eruption syndrome with eosinophilia and systemic symptoms. It has been reported that the typical clinical manifestations of DRESS syndrome induced by vancomycin include extensive rash, fever, eosinophilia, and involvement of multiple organ functions. Among these, rash and fever are the main clinical manifestations [7]. In this study, 13 patients developed fever, which was initially attributed to an infection, but none of them displayed clinical manifestations related to allergy, such as rash.

#### Changes of liver and kidney function and severity of liver injury

Among the 17 patients, yellow skin staining was present in 2 patients (11.76%), with 1 of them having multiple organ failure, while 5 patients (29.41%) experienced fatigue. The remaining ten patients (58.82%) showed no evidence of liver function damage. All 17 patients had abnormal liver function indexes, mainly including increased AST/ALT and increased direct/indirect bilirubin levels. All 17 patients had abnormal AST/ALT levels, with 14 patients (82.35%) having both elevated AST and ALT levels, only 2 patients had an elevated AST level, and 1 patient had an elevated ALT level. In total, nine patients (52.94%) showed abnormal bilirubin levels, with four cases (23.53%) displaying abnormal AST/ALT and bilirubin levels. Among the 17 patients, 7 patients (41.18%) had AST/ALT ≤ 3 upper limit of normal (ULN), while 2 patients (11.76%) had 3 ULN < AST/ALT ≤ 5 ULN, 7 patients (41.18%) had 5 ULN < AST/ALT ≤ 20 ULN, and 1 patient (5.88%) had ≥ 20 ULN.

Among these 17 patients, 5 patients (29.41%) had increased creatinine levels, 3 patients had AST/ALT between 5 ULN and 20 ULN, and 1 patient had AST/ALT above 20 ULN. In total, three of these patients had grade 1 liver injury and one had grade 3 liver injury with an AST/ALT ratio between 5 ULN and 20 ULN. There was no correlation between the severity of liver function injury and increased creatinine level. According to the grading standard of the severity of drug-induced liver injury [8], the liver function of the patients was evaluated, revealing that the severity of liver injury in 15 patients (88.24%) was grade 1; 2 patients with grade 3–4 liver injury showed systemic skin jaundice. Only one patient with vancomycin blood concentration beyond the normal range had grade 4 liver function injury (Table 4).

**Table 4:** Test index anomaly combination

ALT (*n* = 15)	AST (*n* = 16)	Bilirubin (*n* = 9)	Creatinine (*n* = 5)	Cases (*n* = 17)
Abnormal				1
Abnormal	Abnormal			1
Abnormal	Abnormal	Abnormal	Abnormal	4
	Abnormal	Abnormal	Abnormal	1
Abnormal	Abnormal			6
Abnormal	Abnormal	Abnormal		3
	Abnormal	Abnormal		1

### Interventions and outcomes

Among the 17 patients, 1 patient did not receive any intervention, 4 patients stopped taking vancomycin after experiencing liver injury, 1 patient was given a reduction in vancomycin dosage, and 11 patients (64.7%) were treated with hepatic protectants. Among the patients receiving treatment, seven patients were treated with reduced glutathione, three patients were treated with two kinds of hepatic protectants (phosphatidylcholine + adenosylmethionine, magnesium isoglycyrrhizinate + reduced glutathione, reduced glutathione + polyene phosphatidylcholine), one patient was treated with three kinds of hepatic protectants (reduced glutathione, enephosphatidylcholine, and adenosylmethionine). Overall, 11 patients (64.71%) were treated for 3–29 days, with an average of 12.27 ± 9.76 days, and 12 patients (70.59%) showed improvement in liver function after intervention. Among them, eight patients (47.06%) returned to the normal level of liver function index and four patients (23.53%) had continued to decline, but did not return to the normal level during hospitalization. In total, two patients (11.76%) were discharged upon request by their family members without any signs of improvement, with no follow-up data available, and three patients (17.65%) died owing to multiple organ failure caused by the progression of the primary disease, including two patients with respiratory failure and one patient with acute cardiac failure.

## Discussion

When administered intravenously, vancomycin can be distributed through most of the body’s tissues and fluids. It can achieve effective bacterial concentrations in serum, pleural fluid, pericardial fluid, ascites, urine, and atrium, but not in bile. At the same time, the medication is not metabolized in the body and is ultimately excreted in the urine as a prototype. In patients with kidney dysfunction, vancomycin excretion will be mitigated, and studies have shown that abnormal liver function affects the pharmacokinetics of vancomycin. Previous pharmacokinetic studies have shown levels of vancomycin in liver tissue and bile to be below the detection limit [5]. In a Japanese study, vancomycin-conjugated monoclonal antibodies were used to target vancomycin molecules in the kidney and liver of rats, and immunohistochemistry was used to monitor the uptake of vancomycin in the kidney and liver of rats. The study showed that vancomycin is not metabolized by the liver [9].

Compared with nephrotoxicity, even though the correlation between hepatotoxicity and vancomycin was low, abnormal markers of liver function, such as AST, ALT, elevated AFP, and jaundice, were associated with the use of vancomycin. Results of a meta-analysis of 20 RCTs conducted between 1950 and 2010 showed that patients who received vancomycin had a significantly higher incidence of abnormal liver function, especially serum transaminases, compared with patients who did not receive vancomycin. Although the levels are elevated, most are mild to moderate [6]. In a retrospective cohort study of patients receiving vancomycin with hepatic impairment, it was observed that 237 patients with no or mild hepatic impairment and 171 patients with moderate-to-severe hepatic impairment were observed. Patients with hepatic impairment had very high mean concentrations, lower clearance rates, longer half-lives, and higher rates of acute renal damage [10]. Another study demonstrated the significant impact of liver function on vancomycin pharmacokinetics [11] and showed that patients with acute or chronic liver failure are at a higher risk of reduced survival rates [12]. At present, the cause of abnormal liver function indices caused by vancomycin remains unclear; however, timely use of regular doses of vancomycin in patients with liver damage may result in increased blood concentrations [13]. Studies have shown that higher intensive care unit (ICU) mortality rates are observed when the average plasma concentration of vancomycin is 15–20 mg/L or > 20 mg/L [14], and greater incidences of nephrotoxicity are observed when the trough concentration of vancomycin is > 15 mg/L [15]. For the Chinese population, the guidelines recommend that the plasma concentration of vancomycin in adult patients be no higher than 20 mg/L [16]. In this study, through a detailed retrospective analysis of the case data of patients with vancomycin-induced hepatotoxicity, the characteristics of vancomycin hepatotoxicity, including occurrence time, blood concentration, intervention measures, and liver function indicators, were described in detail. In this study, 0.37% of the patients had liver function impairment. Consistent with previous findings, most patients had mild liver injury, mainly characterized by mild to moderate transaminase abnormalities; however, no correlation was observed between the severity of liver injury and blood drug concentration or kidney injury. To confirm whether there was a correlation, the correlation between the time of administration of vancomycin, the total dose, and the blood level was assessed in 17 patients. The results showed that there was no correlation between the increase in liver function indices and the administration time, total dose, and blood concentration of vancomycin, which may be related to the small sample size. Further work is required.

Similar to nephrotoxicity, the mechanism of hepatoxicity is also linked to oxidative stress and mitochondrial malignant lesions [17]. The reason for the large difference between vancomycin nephrotoxicity and hepatotoxicity may be related to the different types of transporters on liver cells and renal tubular cells. Vancomycin nephrototoxicity primarily causes damage to kidney cells by affecting renal tubular mitochondria. While the majority of drug delivery to mitochondria requires transmembrane transport proteins. Recent studies have shown that the expression of *Oat1/3 *and* Oct2* was observed in the rat vancomycin renal injury model, which may play an important role in the transport of vancomycin to the renal proximal tubule cells. At the same time, vancomycin can inhibit the expression of oats 1, oats 3, and oct2, leading to decreased transport of endogenous toxins and increased kidney injury [18]. In the liver, members of the organic anion transporter (OAT) family involved in vancomycin transport are less expressed, which can be the main cause of nephrotoxicity and hepatotoxicity. At the same time, studies have shown that vancomycin can be detected in the hepatic sinusoidal endothelial cells of the liver for up to 8 days after injection, while it was not detected on the surface of hepatocytes and bile capillaries with the therapeutic dose of vancomycin in rats, indicating that vancomycin cannot pass through cells and enter the liver [9]. The study also demonstrates that the vancomycin-bound carrier is absent from the surface of the hepatic cell membrane. At the same time, some studies have shown that the genes of oats and OCT are expressed in certain rat hepatocytes [19], which can be related to the onset of vancomycin hepatotoxicity.

During this study, 17 patients were assessed for liver damage caused by the use of vancomycin. The general condition, severity of liver injury, liver function indices (AST/ALT, direct bilirubin/indirect bilirubin), and liver injury were assessed. The correlation between the severity and vancomycin was analyzed. In all patients, the type of liver injury was mainly asymptomatic abnormal liver function indicators, two patients had jaundice and eventually died of systemic multi-organ failure due to concurrent disease progression. The dosage, time, and blood concentration were analyzed and nine patients (52.94%) had abnormal liver function when they initially used vancomycin with a conventional dose of 1 g every 12 hours. Most of the patients had one to three complications and were necessarily treated with two to four drugs at the same time, including antibacterial drugs, antiviral drugs, antihypertensive drugs, and a few patients were treated with antitumor drugs, which may exacerbate hepatotoxicity and severe liver injury in patients who used two drugs. Overall, 94.1% of patients reported abnormal liver function within 7 days of taking vancomycin. In 76.47% of patients, the blood concentration was out of the range of effective blood concentration. Among the seven patients who exceeded the normal value, the Naranjo’s score of 85.71% patients was probably related, suggesting that higher blood concentration may lead to the occurrence of abnormal liver function, but no linear relationship between blood concentration and liver injury was found. In this study, the hepatic toxicity induced by vancomycin was described in detail, and patient data were analyzed to provide a baseline for the clinical use of vancomycin. However, owing to the small amount of data and large patient differences, more data are needed for more in-depth analysis.

## Conclusion

In the clinical setting, the focus should be on the liver toxicity of vancomycin, and the liver function indicators of patients should be monitored.

## Data Availability

All data generated or analyzed during this study are included in this published article.
